# RETRACTION: Comprehensive Analysis of Risk Factors for Surgical Site Infections Following Thoracoscopic Radical Resection in Patients With Lung Cancer

**DOI:** 10.1111/iwj.70593

**Published:** 2025-04-21

## Abstract

**RETRACTION:** TaoT. , LiQ. , YangY. , and WangG. , “Comprehensive Analysis of Risk Factors for Surgical Site Infections Following Thoracoscopic Radical Resection in Patients With Lung Cancer,” International Wound Journal 21, no. 3 (2024): e14525, 10.1111/iwj.14525.38013589 PMC10898367

The above article, published online on 27 November 2023, in Wiley Online Library (http://onlinelibrary.wiley.com/), has been retracted by agreement between the journal Editor in Chief, Professor Keith Harding; and John Wiley & Sons Ltd. Following an investigation by the publisher, all parties have concluded that this article was accepted solely on the basis of a compromised peer review process. The editors have therefore decided to retract the article. The authors did not respond to our notice regarding the retraction.

**RETRACTION:** T. Tao , Q. Li , Y. Yang , and G. Wang , “Comprehensive Analysis of Risk Factors for Surgical Site Infections Following Thoracoscopic Radical Resection in Patients With Lung Cancer,” International Wound Journal 21, no. 3 (2024): e14525, 10.1111/iwj.14525.38013589 PMC10898367

The above article, published online on 27 November 2023, in Wiley Online Library (http://onlinelibrary.wiley.com/), has been retracted by agreement between the journal Editor in Chief, Professor Keith Harding; and John Wiley & Sons Ltd. Following an investigation by the publisher, all parties have concluded that this article was accepted solely on the basis of a compromised peer review process. The editors have therefore decided to retract the article. The authors did not respond to our notice regarding the retraction.

